# An object-identity probability cueing paradigm during grasping observation: the facilitating effect is present only when the observed kinematics is suitable for the cued object

**DOI:** 10.3389/fpsyg.2015.01479

**Published:** 2015-09-29

**Authors:** Laila Craighero, Sonia Mele, Valentina Zorzi

**Affiliations:** Section of Human Physiology, Department of Biomedical and Specialty Surgical Sciences, University of Ferrara, Ferrara, Italy

**Keywords:** attention to objects, action observation, simple reaction times, affordances, premotor theory of attention, action prediction

## Abstract

Electrophysiological and psychophysical data indicate that grasping observation automatically orients attention toward the incoming interactions between the actor’s hand and the object. The aim of the present study was to clarify if this effect facilitates the detection of a graspable object with the observed action as compared to an ungraspable one. We submitted participants to an object-identity probability cueing experiment in which the two possible targets were of the same dimensions but one of them presented sharp tips at one extreme while the other presented flat faces. At the beginning of each trial the most probable target was briefly shown. After a variable interval, at the same position, the same (75%) or a different target (25%) was presented. Participants had to press a key in response to target appearance. Superimposed to the video showing cue and target, an agent performing the reaching and grasping of the target was presented. The kinematics of the action was or was not suitable for grasping the cued target, according to the absence or presence of the sharp tips. Results showed that response was modulated by the probability of target identity but only when the observed kinematics was suitable to grasp the attended target. A further experiment clarified that response modulation was never present when the superimposed video always showed the agent at a rest position. These findings are discussed at the light of neurophysiological and psychophysical literature, considering the relationship between the motor system and the perception of objects and of others’ actions. We conclude that the prediction of the mechanical events that arise from the interactions between the hand and the attended object is at the basis of the capability to select a graspable object in space.

## Introduction

Selective attention is the name given to the capability of selecting a particular stimulus according to its physical properties and way of presentation, or to previous contingencies and instructions. After selection, the stimulus is processed and, if convenient for the individual, acted on. The premotor theory of attention (Rizzolatti et al., 1994; Rizzolatti and Craighero, 1998) claims that selective attention derives from mechanisms that are intrinsic to the circuits underlying perception and action. This account is deeply rooted in neurophysiological findings on how space is coded and transformed into action in the nervous system. In primates, space is coded in a series of parietofrontal circuits working in parallel (Rizzolatti et al., 1997; Matelli and Luppino, 2001; Craighero, 2014). The activation of those cortical circuits and subcortical centers, involved in the transformation of spatial information into action, determines both an increase in the motor readiness to respond to a specific space sector and a facilitation of processing stimuli coming from that space sector (Moore and Fallah, 2001). The main assumption of the premotor theory is that the motor programs for acting in space, once prepared, are not immediately executed. The condition in which action is ready but not executed corresponds to what is introspectively called spatial attention orienting (Rizzolatti and Craighero, 1998). The premotor theory of attention has received support from electrophysiological (Moore and Armstrong, 2003; Ruff et al., 2006; Ekstrom et al., 2008) and brain imaging (Corbetta et al., 1998; Nobre et al., 2000) studies and has been extended from spatial attention to attention directed to objects. In particular, there is evidence that preparation to act on an object produces faster processing of stimuli congruent with that object (Craighero et al., 1999; Bekkering and Neggers, 2002; Fischer and Hoellen, 2004; Hannus et al., 2005; Fagioli et al., 2007; Symes et al., 2008).

However, many experimental evidences have been collected to prove the presence of a representational sharing between action execution and action observation, particularly evident in the phenomenon named motor resonance, in which the observer’s motor system is dynamically (online) replicating the observed movements (Fadiga et al., 1995; Brighina et al., 2000; Gangitano et al., 2001; Clark et al., 2004; Montagna et al., 2005; Borroni and Baldissera, 2008). In other words, an observed action is subliminally reenacted, which exactly corresponds to the condition known as attention orienting. An evidence of the attentional consequence induced by action observation is provided by the presence of proactive gaze behavior during observation of a block stacking task (Flanagan and Johansson, 2003) indicating that the observers’ gaze, and therefore their attention, is not following the hand’s trajectory but it is focused onto the goal of the action well before the arrival of the actor’s hand. This result is usually explained as a consequence of the fact that each observed action is mapped onto the sensorimotor representation of that same action, allowing one to understand its meaning and to predict its outcome (Rizzolatti and Craighero, 2004). Since in visually guided actions, for planning and control purposes, gaze usually leads the hand to objects to-be-grasped, one may hypothesize that the same proactive gaze behavior is present in action observation. In a recent experiment, Flanagan et al. (2013) showed that this gaze behavior is more likely deputed to evaluate the mechanical events that arise from interactions between the actor’s hand and objects, than to predict the target object of the actor’s reaching movement. Mechanical events mark transitions between consecutive action phases and represent sub-goals of the overall planning and control of manipulation tasks. For example, when lifting, contact between the digits and object marks completion of the reach. Indeed, a series of experiments investigating the capacity to detect this time-to-contact showed that during action observation the exact instant at which a grasping hand touches an object is faster detected when grasping action’s kinematic parameters correspond to those predicted by the observer on the basis of contextual information, in particular the suitability of the observed actions in grasping the object (Craighero et al., 2008; Craighero and Zorzi, 2012). Specifically, it has been demonstrated that, on equal terms of kinematics, time-to-contact detection times were delayed, and motor evoked potentials from hand intrinsic muscles were reduced, when the movement shown was not suitable to grasp the object (Craighero et al., 2014). All these data suggest that grasping observation automatically orients attention toward the incoming interactions between the actor’s hand and the object.

To better understand the characteristics of this effect, the present experiment aimed to verify if observing an agent executing a grasping action induces a faster detection of the to-be-grasped object. To this end, we designed an object-identity probability cueing experiment and we superimposed the video of an agent executing an action suitable or not suitable to grasp the cued object. In particular, Experiment 1 consisted in a simple reaction time (RT) experiment in which participants had to press a key at the appearance of one of two possible graspable targets always presented at the same position. The probability regarding the identity of the target was given by a preceding cue, showed at the same position, representing one of the two targets: in 60% of the trials the target was the same as the cue, and in 20% of the trials the target was a different one. In the remaining 20% of the trials the target did not appear. Superimposed to the video showing cue and target, an agent performing the reaching and grasp action suitable for only one of the two targets was presented. Consequently, we included an experimental manipulation such that the kinematics of the action was or was not suitable for grasping the cued target. It is necessary to underline that the identity of the target as well as the movement executed by the agent were absolutely irrelevant for the task execution.

In order to exclude an influence of kinematics induced by merely visual and non-motor-related characteristics, we needed a movement which was visually suitable to grasp both objects. For this reason, trajectory and maximum grip aperture had to be adequate for both objects and, consequently, the two objects should have the same size. However, to have a movement with apparent adequate kinematics but not suitable to grasp one of the two objects, we had to manipulate the intrinsic characteristics of one of them, such as slippery, extreme temperature, presence of sharp tips, etc., rendering it ungraspable with that movement. Given the fact that the characteristics slippery and extreme temperature are not visually perceivable, we decided to use sharp tips. Therefore, we presented a bar and a three-dimensional object with flat faces and sharp tips. The two objects had the same dimensions, and the tips were positioned in correspondence of fingers’ opposition space. To be sure that participants were aware of grasping unsuitability, they were required to exactly replicate the observed movement toward the two real objects before the experiment started. The weight of the object was sufficiently great (74 g) to prevent sharp tip object grasping since the result was quite painful. In contrast, grasping and lifting the flat object was really easy.

Since simple RT experiments investigating the possibility to influence object-identity expectation by manipulating its probability are not present in the literature, we replicated Experiment 1 but the superimposed video always showed the agent at a rest position. Therefore, Experiment 2 was designed to investigate the effects of object-identity expectation on object detection in order to verify the possible influence that this manipulation has on the results of Experiment 1.

## Experiment 1

### Materials and Methods

#### Participants

Thirty-six students (aged 20–27, 18 females) of the University of Ferrara served as participants. All were right-handed according to the Edinburgh Handedness Inventory (Oldfield, 1971), had normal or corrected-to-normal visual acuity, were unaware of the purposes of the study and were debriefed at the end of the experimental session. The procedures of the study were approved by the local ethical committee.

#### Stimuli and Procedure

Participants were seated in front of a desk and watched the videos presented on a 60 Hz CRT monitor (resolution 1024 × 768 pixels) placed at 60 cm distance. The videos showed an agent executing a reaching and grasping action toward an object. The object (Cue) was present on the desk before the start of the action, it disappeared during the execution of the reaching and then the same or a different object (Target) reappeared in the same position at the instant of touch. Participants were instructed to observe the Cue object and to tap with their right index finger on a conducting pad placed on the table at the instant at which the Target object appeared (experimental trials), and to refrain to tap when the object did not reappear (catch trials). Participants’ left arm was maintained relaxed on the arm rest.

**FIGURE 1:**
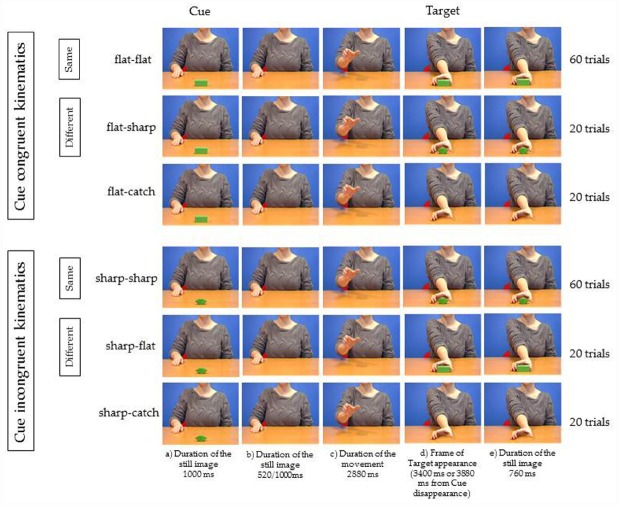
**Experiment 1.** Five frames relative to each of the six videos used as stimuli. The object shown at step (a) for 1000 ms is the Cue and represents the Target appearing in 60% of the trials (Same trials). In 20% of the trials the uncued Target was presented (Different trials). In 20% of the trials the Target was not presented. Participants had to respond by tapping with their right index finger on a conducting pad placed on the table when Target object appeared (flat–flat, flat–sharp, sharp–sharp, sharp–flat), and to refrain to tap when the object did not reappear (flat-catch, sharp-catch). In one session the kinematics of the agent was suitable to grasp the cued object (Cue congruent kinematics). In the other session the kinematics of the agent was not suitable to grasp the cued object (Cue incongruent kinematics). See text for more details.

Six possible videos (Figure 1) were presented. To produce the videos we followed the following procedure.

**FIGURE 2:**
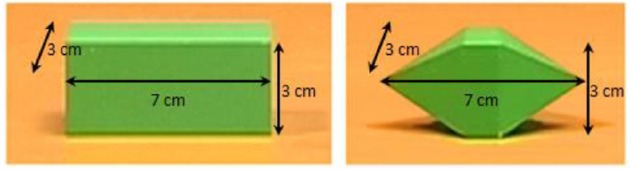
**The two objects presented in the videos.** On the left, the object grasped by the experimenter in the original video (flat–flat video). On the right, the object artificially introduced via software as Cue (sharp–flat and sharp-catch videos), as Target (flat–sharp video), and as Cue and Target (sharp–sharp video).

We first videotaped one video in which the agent was sitting at a desk. A bar (7 cm length, 3 cm width, 3 cm height; Figure 2, left) was located at a 60 cm distance in front of the agent’s chest at the center of the body’s midline. The bar was placed with its longer axis facing the agent. The agent reached and grasped the bar with natural velocity with fingers’ opposition space parallel to the frontal plane, without lifting the object.

Then we defragmented the video saving each frame as bitmap image (25 frames/second; duration of 1 frame = 40 ms; frame size: 720 × 576 pixels) and we manipulated the frames in order to obtain the following sequence for the “flat–flat video” as follows:

(a) for 1000 ms, presentation of the model with the right hand on the desk behind the object;
(b) using Adobe Photoshop 7.0, the object was canceled and only the model was presented for 520 ms (13 frames: 13 × 40 ms = 520 ms) in half the trials, or for 1000 ms (25 frames: 25 × 40 ms = 1000 ms) in the other half of the trials;
(c) the model was shown executing the reaching action in the absence of the object (2880 ms duration);
(d) at the end of the reaching action (after 3400 or 3880 ms from the disappearance of the object) the object was shown again.
(e) the object was shown for 760 ms.

To obtain the “sharp–sharp video” we captured an image (saved as bitmap image; frame size: 720 × 576 pixels) of a three-dimensional object with flat faces and sharp tips, of the same dimensions as the bar (7 cm length, 3 cm width, 3 cm height; Figure 2, right), placed on the desk exactly in the same position as the bar during videotaping, and we extrapolated the object using Adobe Photoshop 7.0. In each frame of the flat object video in which the object was present we substituted the bar with the object with sharp tips and saved the frames reworked as bitmap images.

To obtain the catch videos (“flat-catch,” “sharp-catch”), we replicated the sequence for both object videos but at point (d) of the sequence the object was not shown.

To obtain the “flat–sharp” and the “sharp–flat” videos, we replicated the sequence for both object videos but at point (d) and (e) of the sequence of the flat object video we presented the object with sharp tips (flat–sharp) and at point (d) and (e) of the sequence of sharp tip object video we presented the bar (sharp–flat).

Using the frames of the different categories of stimuli, we edited the six videos by means of Adobe Premiere Pro 1.5. Total duration of the videos: 5160 ms in half the trials or 5640 ms in the other half of the trials.

The six videos differed for two experimental manipulations. One manipulation was the identity between the Cue object and the Target object (“Identity”). The two objects were the same in 60% of the experimental trials when flat–flat and sharp–sharp videos were presented (same trials) and different in 20% of the experimental trials when flat–sharp and sharp–flat videos were presented (different trials). The other manipulation was the congruency between the kinematics of the observed movement and the intrinsic properties of the Cue object (“Kinematics congruence”). They were congruent in 50% of the experimental trials when flat–flat videos and flat–sharp videos were presented and they were incongruent in 50% of the experimental trials when sharp–sharp videos and sharp–flat videos were presented.

To ensure the highest temporal resolution between the presentations of the frame indicated at the (d) instant of each video (appearance of the Target) and the response given by the participants, a light sensor was placed at the bottom right of the monitor surface. In correspondence of it, a square (150 × 150 pixels) was inserted in each frame of the experimental videos. This square was black and turned white at the (d) instant of each video. The time lag between the signal recorded by the conducting pad used by the participant to respond and the change of brightness of the square was used as dependent variable. Errors were considered those trials in which the response preceded or followed the agent’s touch of at least 800 ms, and trials with errors were resubmitted to the participant.

To make participants aware of the suitability of the observed grasping, before the experiment they were asked to try to grasp and lift the two objects once, by using the same finger opposition space used in the videos. Grasping the sharp tip object by having the two tips in correspondence of fingers’ opposition space was impossible as it was quite painful. In contrast, grasping and lifting the flat object was very easy. Afterward they completed a training block of 10 trials in which all videos were presented to familiarize them with the experimental stimuli.

Participants were submitted to 200 trials: 60 trials for both flat–flat and sharp–sharp videos; 20 trials for both flat–sharp and sharp–flat videos; 20 trials for both catch-flat and catch-sharp videos.

The experiment was subdivided into two sessions, each consisting of 100 trials. Each session differed for the Cue object. The order of sessions was counterbalanced between participants and the second session was executed after a brief rest period. The trials were randomized within each session.

#### Data Analysis

Mean RTs of responses were used for the analysis. Data were entered into and analyzed by two-way repeated measures analysis of variance (ANOVA). The study used a 2 × 2 design with two repeated-measures variables: Kinematics congruence (congruent versus incongruent) and Identity (same versus different). All pairwise comparisons were performed using the Bonferroni *post hoc* test. A significance threshold of *P* < 0.05 was set for all statistical analyses. Effect sizes were estimated using the partial eta square measure (${\text{η}}_{p}^{2}$). The data are reported as mean ± standard error of the mean (SEM).

### Results

**FIGURE 3:**
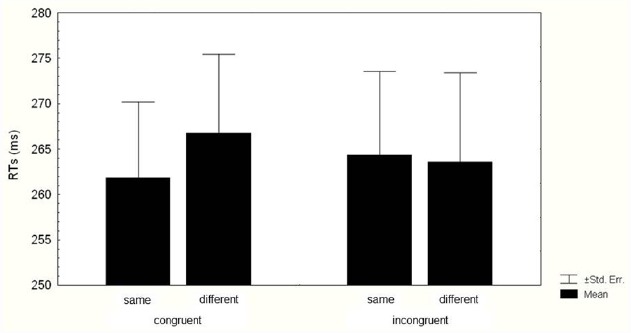
**Mean reaction times (RTs) of responses.** Data for valid and invalid trials when the kinematics of the agent was suitable (congruent kinematics) and when it was not suitable (incongruent kinematics) to grasp the cued target are shown. Thin lines above histograms indicate standard error of the mean. Ordinates are in milliseconds.

The two-way ANOVA on RTs revealed a significant main effect of Identity (*F*_1,35_ = 4.190, *p* = 0.048, ${\text{η}}_{p}^{2}$ = 0.106), because RTs were faster in same trials (263.08 ± 8.59 ms) than in different trials (265.17 ± 9 ms). The two-way interaction Kinematics congruence × Identity (*F*_1,35_ = 5.197, *p* = 0.028, ${\text{η}}_{p}^{2}$ = 0.129) was also significant (Figure 3). Bonferroni *post hoc* analyses indicated that when the kinematics was congruent with the Cue, RTs were faster in same trials (261.83 ± 8.36 ms) than in different trials (266.72 ± 8.67 ms; *p* = 0.04). When the kinematics was incongruent with the Cue, the Identity effect did not occur, indeed RTs in same trials (264.33 ± 9.23 ms) did not differ from RTs in different ones (263.61 ± 9.8 ms; *p* = 1).

The number of error trials was irrelevant. Furthermore, almost all the errors detected were generally due to temporary problems in the conducting pad. Responses given during catch trials were almost absent (at most two errors for each participant).

## Experiment 2

The results of Experiment 1 indicated that the facilitating effect determined by the expectation of the cued target was present only when the observed kinematics was suitable to grasp it. However, given the impossibility to design an experiment considering the counterbalance between the two types of kinematics (absence of two objects and two types of grasping able to satisfy in an orthogonal way all the experimental requirements), it is not possible to exclude that the absence of the facilitating effect for the sharp object was determined by the object itself and not by the observation of an incongruent kinematics. To solve this question, and to verify if mere object-identity expectation is able to influence object detection RTs, we replicated Experiment 1 but the superimposed video always showed the agent at a rest position.

### Materials and Methods

#### Participants

A new group of 20 students (10 females) of the University of Ferrara (mean age = 24.45 years, standard deviation = 3.48) participated in the experiment. All participants except two were right-handed. Participants were unaware of the purposes of the study and were debriefed at the end of the experimental session.

#### Stimuli and Procedure

Before the experiment participants were required to grasp and lift the two real objects with the same grasping used in Experiment 1. Therefore, no difference in motor experience was present between the two groups of participants.

The only difference between the two experiments regarded the stimuli: the video showing the grasping in Experiment 1 was always substituted by the still image of the agent at a rest position as in step (a) of Figure 1.

**FIGURE 4:**
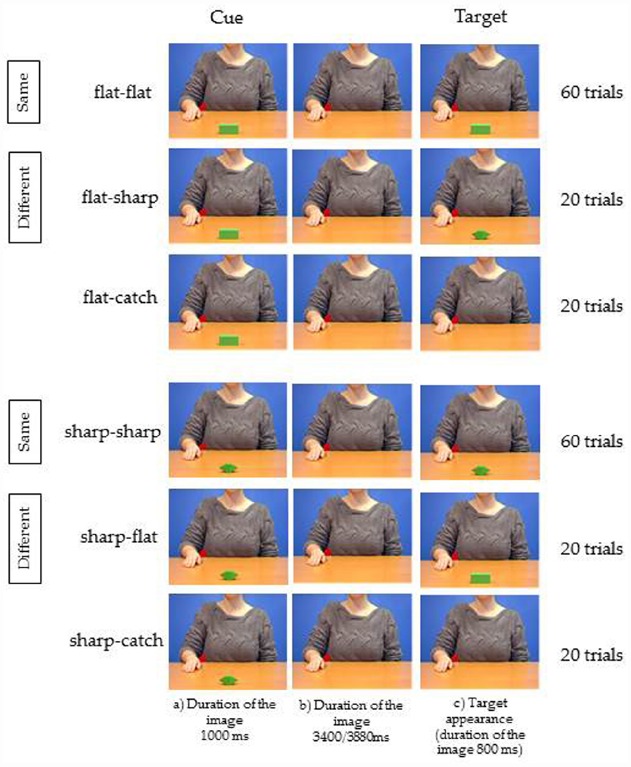
**Experiment 2.** Three frames relative to each of the six videos used as stimuli. The stimuli and procedure are those described in Figure 1. The only difference consisted in the fact that the agent shown in the background was not moving, as in Experiment 1, but she was always presented at a rest position as in step (a) of Figure 1.

The instructions, the sequence of events, the number of trials, the time of target presentation and the type of response, were exactly the same as in Experiment 1, apart from the absence of the condition Kinematics congruence (Figure 4).

#### Data Analysis

Mean RTs of responses were used for the analysis. Data were coded on the basis of the type of Cue (flat, sharp) and the identity between the Cue and the Target (same, different). Data were entered into and analyzed by two-way repeated-measures ANOVAs. The study used a 2 × 2 design with two repeated-measures variables: Cue (flat, sharp) and Identity (same or different). All pairwise comparisons were performed using the Bonferroni *post hoc* test. A significant threshold of *p* < 0.05 was set for all statistical analyses. Effect sizes were estimated using the partial eta square measure (${\text{η}}_{p}^{2}$). The data are reported as mean ± SEM.

### Results

The two-way ANOVA on RTs revealed that the main effect of Cue (*F*_1,19_ = 0.241, *p* = 0.628, ${\text{η}}_{p}^{2}$ = 0.012), the main effect of Identity (*F*_1,19_ = 0.704, *p* = 0.411, ${\text{η}}_{p}^{2}$ = 0.035), and the two-way interaction Cue × Identity (*F*_1,19_ = 2.452, *p* = 0.133, ${\text{η}}_{p}^{2}$ = 0.114) were not statistically significant (Flat Cue: same Target, 368.43 ± 9.7 ms; different Target, 366.29 ± 9.45 ms. Sharp Cue: same Target 359.64 ± 8.60 ms; different Target, 367.3150 ± 7.9 ms).

The number of error trials and of catch trials was irrelevant.

Therefore, the present results indicate that, relatively to the present experimental design, mere object-identity expectation is not able to influence object detection RTs and, consequently, we can exclude that the absence of the facilitating effect for the sharp object found in Experiment 1 was determined by the object itself. It is to note that RTs are clearly slower than those obtained in Experiment 1. This finding is probably due to an unspecific effect determined by motion observation.

## Discussion

The Premotor theory of attention is an influential idea that experimentally rejected the information processing models of attention. These models claim that information enters the sensory or motor system and is then relayed into memory via an attentional mechanism that is independent of the sensory and motor system, conceiving it as a modular, “higher” cognitive function. On the contrary, according to the Premotor theory of attention, spatial attention and attention to objects are the consequence of activation of the motor system, and shifts of attention are achieved by planning goal-directed actions, such as eye-movements and reaches, according to the cues present in the environment. A series of studies investigating the presence of a representational sharing between action execution and action observation indicate that grasping observation automatically orients attention toward the mechanical events that arise from interactions between the actor’s hand and objects (Craighero et al., 2008; Craighero and Zorzi, 2012; Flanagan et al., 2013). To deeply understand this phenomenon, the present study aimed to verify if observing an agent executing a grasping action induces a faster detection of the to-be-grasped object. We submitted participants to a simple RT task in which the target appeared always at the same position but its identity changed with a fixed 75–25% ratio, according to a cue presented at the beginning of each trial. Superimposed to the cue-target presentation, an agent performing a reaching-grasping action toward the target was presented. The target appeared at the time-to-contact of the hand grasping the target. However, the kinematics of the observed action was suitable to grasp only one of the two possible targets. Participants were aware of the suitability of the observed grasping because, before the experiment, they were asked to try to grasp and lift the two objects once by using the same finger opposition space used in the videos. This experience allowed them to know that grasping the sharp tip object in this way was not possible. Results showed that the irrelevant observed movement, characterized by a kinematics incongruent with the to-be-grasped object, dramatically influenced the results, canceling the facilitating effect induced by the cue that was present when the kinematics was congruent with the object. The results of Experiment 2, in which the agent was presented always at the rest position, showed the absence of a facilitating effect, independently from the identity of the cued object. These data excluded the possibility that the difference between the two kinematics conditions was determined by specific characteristics of the objects themselves. Furthermore, they excluded both the possibility to influence object-identity expectation by manipulating its probability, and the presence of an effect of priming, when tested in a simple RT experiment. The priming effect, i.e., the notion that merely attending to a feature enhances the processing of that feature across the visual field, may occur in an automatic bottom up way (Theeuwes, 2013). This priming may have a mere sensory origin: Target selection derives from the setting up of a target template that needs to be matched to a sensory signal (von Wright, 1970). Alternatively, priming may have a sensorimotor origin. In accord with neurophysiological (Di Pellegrino et al., 1992; Gallese et al., 1996; Martin et al., 1996; Rizzolatti et al., 1996; Grafton et al., 1997; Chao and Martin, 2000; Gerlach et al., 2002; Grezes and Decety, 2002; Grezes et al., 2003; Carpaneto et al., 2011) and psychophysical (Klatzky et al., 1995; Craighero et al., 1996, 1998; Tucker and Ellis, 1998, 2001, 2004; Ellis and Tucker, 2000) evidence, seeing an object facilitates an action congruent with the visual properties of that same object. However, none of the cited works used a simple RT experiment with no comparison between responses given by the two hands (i.e., one hand can be more suitable than the other in interacting with the to-be-responded object; see Tucker and Ellis, 1998).

### The Facilitating Effect is Modulated by the Observed Action Kinematics

The main result of the present study is that simple RTs recorded in response to the detection of the target object were faster when the target corresponded to the cue indicating the higher probability of appearance of the target, but only when the kinematics of the reaching and grasping action presented in the background, and irrelevant for the task, was suitable to grasp the cued object. In light of the results of Experiment 2, it is necessary, therefore, to consider a possible influence of observed action kinematics on task execution. A recent experiment indicated that during grasping observation, time-to-contact detection times were delayed, and motor evoked potentials recorded in hand intrinsic muscles were reduced, when the observed movement was not suitable to grasp the object (Craighero et al., 2014). These results are interpreted as evidence that during grasping observation the motor system of the observer is automatically influenced both by the seen movements and by the intrinsic properties of the to-be-grasped object. According to this possibility, we can assume that the vision of the sharp object as cue automatically activates the sensorimotor representation of the only possible action suited to grasp the object, which is to grasp it along its shorter axis which does not present sharp tips (sagittal grasping). However, as soon as the observed movement begins, it activates the sensorimotor representation in the observer relative to the grasping along the object’s longer axis (parallel grasping). Therefore, in this cue incongruent kinematics condition, the action representation activated by the to-be-grasped object (sagittal grasping) and the one activated by the observed movement (parallel grasping) do not coincide. This incongruence may determine a reduction in the excitability of the underlying motor circuits, causing a decrease in the ability to perceive related visual stimuli (Borgomaneri et al., 2015). This effect may explain the absence of the facilitating effect. This possibility is supported by the experimental evidence that motor evoked potentials, recorded during observation of a grasping movement in which the kinematics is not suitable to grasp the to-be-grasped object, did not differ from those recorded during the control condition (Craighero et al., 2014). These results reveal a reduction in corticospinal excitability, indicating the presence of a motor suppression during observation of incongruence between the sensorimotor representation cued by the to-be-grasped object and that cued by the observed action. This finding is in accord with other results present in literature (Gangitano et al., 2004; D’Ausilio et al., 2011) suggesting that the proactive role of the motor system, able to anticipate the consequences of actions on the basis of contextual cues, and acting as an online feedback-based control strategy, stops whenever the activated motor plan ceases to match the attended one, probably reflecting the necessity to make the necessary corrections. Already, Lotze (1852) suggested the possibility that perception involves an anticipation of the relevant action by arguing that the organization of sensorial data is the outcome of its integration with information gleaned from the muscles, and Janet (1935), ranked alongside William James and Wilhelm Wundt as one of the founding fathers of psychology, also claimed the predictive nature of perception by proposing that our brain formulates hypothesis for movement, predisposing the action best suited to the situation, prior to making any movement. People may use internal predictive models to provide sensory expectations that are used to monitor and control goal-directed actions (Desmurget and Grafton, 2000). Analogously, it has been argued that the same internal modeling mechanisms are reused when we encode another’s action in terms of our own motor repertoire (Fadiga et al., 1995; Gallese et al., 1996; Rizzolatti and Craighero, 2004; Fazio et al., 2009). In this sense, the motor system might furnish an attentional-like mechanism able to prime perceptual processes (Rizzolatti et al., 1987, 1994; Rizzolatti and Craighero, 1998).

In the condition in which the kinematics of the movement is suitable to grasp the cued object, the incongruence between sensorimotor representations is not present and therefore the motor system may fully contribute to the prediction of the sensory expectation regarding the target object. The cue activates the related grasping movement representation or, alternatively, all the possible related grasping movement representations. Specifically, both the sagittal and the parallel grasping are suitable to grasp the flat object. In this case, parallel grasping is consistent with the observed action, and the activation of its motor representation reduces the time required to detect the visual object the intrinsic properties of which match those of the cue, as happens in the classical motor-visual attentional effect (Craighero et al., 1999).

### The Facilitating Effect Probably is Not Determined by the Observed Action Kinematics Only

Present data, however, cannot be considered exclusively as an automatic effect of object and action observation. The magnitude of the RTs excludes the possibility that the response given by the participants was solely driven by the temporal dynamics of the observed action or by the internal replica of that action. In time-to-contact experiments, when the instructions to the participants are to respond at the instant at which the agent in the videos touched the to-be-grasped object (Craighero et al., 2008, 2014; Craighero and Zorzi, 2012), results show that subjects’ response times are well below those commonly found in simple RTs tasks, being almost coincident with the instant of touch (e.g., in Craighero et al., 2014; in response to suitable actions observation: mean = –12.37 ms, SEM = 8.94). Mean simple RTs for college-age individuals are about 190 ms for light stimuli and about 160 ms for sound stimuli (Galton, 1899; Fieandt et al., 1956; Brebner and Welford, 1980; Welford, 1980). These findings are in favor of the interpretation that, to accomplish the time-to-contact detection task, participants indeed used a predictive model which allows internally simulating the instant of touch and giving the response accordingly. The magnitude of the present RTs (the total mean is around 264 ms), on the contrary, is more similar to the one usually recorded during the execution of an orienting of attention task (e.g., Posner et al., 1978; the range covered by invalid and valid trials is between 200 and 300 ms) than to the magnitude of simple RTs or of time-to-contact detection times. Consequently, it is likely that this difference is prompted by the fixed 75–25% ratio of target identity probability indicated by the cue. However, further experiments are necessary to verify this possibility. In particular, one possible modification of the experiment could be quite interesting, that is the replica of the experiment including a 50–50% ratio for target identity probability. This condition could be represented by the absence of the cue, or by the appearance of an object neutral regarding the requirements for affordance (e.g., a sphere), or by the same cues used in the present experiment with the explicit instruction to the participants that they do not indicate any difference in the probability of the identity of the target. If RTs are modulated by the explicit probability of appearance of the target, this may be a sufficient demonstration of a “more cognitive” evaluation of the cue.

### Physiological Evidence in Favor of the Interpretation of the Results

The main tenet of the present paper is that during grasping observation the motor system of the observer is automatically influenced both by the seen movements and by the intrinsic properties of the to-be-grasped object. A long series of experiments provides physiological evidence in favor of this possibility. Neuroimaging studies have shown that both the observation of others’ actions (for a meta-analysis see, Caspers et al., 2010) and the observation of manipulable objects (Chao and Martin, 2000; Okada et al., 2000; Grezes and Decety, 2002; Creem-Regehr and Lee, 2005; Noppeney et al., 2005) are associated with activation in motor-related regions. This type of research was prompted by the finding that in monkey ventral premotor area F5 there are visuomotor neurons discharging during the execution of specific motor acts and during the observation of graspable objects (“canonical” neurons; Murata et al., 1997; Raos et al., 2006), or during the observation of others’ grasping actions (“mirror” neurons; Gallese et al., 1996; Rizzolatti et al., 1996). Canonical neurons are deemed to be crucial for the transformations of object physical properties into the most appropriate motor act (Jeannerod, 1995), while the crucial feature of mirror neurons consists of matching the sensory description of an observed act with its corresponding motor representation in the observer’s brain (Rizzolatti and Craighero, 2004). The presence of these two classes of visuomotor neurons is clearly in favor of the idea that motor representations can be activated either by the sight of potential target objects or by the observation of other’s actions. A very recent experiment, however, added a substantial novelty to the way in which the motor system may be activated by an action related-visual stimulus. In fact, Bonini et al. (2014) showed the presence in the same ventral premotor area F5 of a set of neurons showing both canonical and mirror properties, discharging to object presentation as well as during the observation of experimenter’s goal-directed acts (canonical-mirror neurons). The authors argued that these neurons might provide a predictive representation of the impending action of the observed agent. This finding largely simplifies the interpretation of the results of the present paper, by easily explaining why no facilitating effect should be present when the observed grasping action and the to-be-grasped object are incompatible. According to the functional properties of these neurons (they fire both during object observation and during the observation of another individual grasping that object), in fact, it is not necessary to separately discuss the influence of the seen movements and of the intrinsic properties of the to-be-grasped object, since these informations converge on the same neuronal substrate. Only the presence of congruence between the converging information could activate this type of sensorimotor representation.

## Conclusion

The present study investigates for the first time the effects of identity probability cueing on a simple RT task. Results show that, even if the identity of the target is irrelevant for the task, when the target is a graspable object, all the motion cues present in the context suggesting target identity inevitably influence the response. Only when all the cues are congruent, a facilitation in simple RTs is present. These data are in favor of the idea that, during grasping, the motor system works as a feedforward anticipatory mechanism based on contextual cues, in order to predict the mechanical events that arise from interactions between the hand and the object. This mechanism is constantly verifying incoming information and it quiets whenever an incongruence is present, probably reflecting the necessity to make the necessary corrections. The contextual cues influencing the mechanism, however, seem to be not only those determining an automatic involvement of the motor system, such as action observation or the knowledge of the to-be-grasped object intrinsic properties, but also those inducing a voluntary expectation of the target object, such as the experimentally manipulated ratio of target identity probability. This last observation points to what commonly happens in real life during grasping execution, when we actively look for the object we want to grasp, selecting it among the multiple objects present on a cluttered table.

### Conflict of Interest Statement

The authors declare that the research was conducted in the absence of any commercial or financial relationships that could be construed as a potential conflict of interest.

## References

[B1] Bekkering H. Neggers S. F. (2002). Visual search is modulated by action intentions. Psychol. Sci. 13, 370–374. 10.1111/j.0956-7976.2002.00466.x 12137141

[B2] Bonini L. Maranesi M. Livi A. Fogassi L. Rizzolatti G. (2014). Space-dependent representation of objects and other’s action in monkey ventral premotor grasping neurons. J. Neurosci. 34, 4108–4119. 10.1523/JNEUROSCI.4187-13.2014 24623789 PMC6705269

[B3] Borgomaneri S. Gazzola V. Avenanti A. (2015). Transcranial magnetic stimulation reveals two functionally distinct stages of motor cortex involvement during perception of emotional body language. Brain Struct. Funct. 220, 2765–2781. 10.1007/s00429-014-0825-6 25023734 PMC4549387

[B4] Borroni P. Baldissera F. (2008). Activation of motor pathways during observation and execution of hand movements. Soc. Neurosci. 3, 276–288. 10.1080/17470910701515269 18979381

[B5] Brebner J. T. Welford A. T. (1980). “Introduction: an historical background sketch,” in Reaction Times, ed. Welford A. T. (New York: Academic Press), 1–23.

[B6] Brighina F. La Bua V. Oliveri M. Piazza A. Fierro B. (2000). Magnetic stimulation study during observation of motor tasks. J. Neurol. Sci. 174, 122–126. 10.1016/S0022-510X(00)00271-9 10727697

[B7] Carpaneto J. Umiltà M. A. Fogassi L. Murata A. Gallese V. Micera S. (2011). Decoding the activity of grasping neurons recorded from the ventral premotor area F5 of the macaque monkey. Neuroscience 188, 80–94. 10.1016/j.neuroscience.2011.04.062 21575688

[B8] Caspers S. Zilles K. Laird A. R. Eickhoff S. B. (2010). ALE meta-analysis of action observation and imitation in the human brain. Neuroimage 50, 1148–1167. 10.1016/j.neuroimage.2009.12.112 20056149 PMC4981639

[B9] Chao L. L. Martin A. (2000). Representation of manipulable man-made objects in the dorsal stream. Neuroimage 12, 478–484. 10.1006/nimg.2000.0635 10988041

[B10] Clark S. Tremblay F. Ste-Marie D. (2004). Differential modulation of corticospinal excitability during observation, mental imagery and imitation of hand actions. Neuropsychologia 42, 105–112. 10.1016/S0028-3932(03)00144-1 14615080

[B11] Corbetta M. Akbudak E. Conturo T. E. Snyder A. Z. Ollinger J. M. Drury H. A. (1998). A common network of functional areas for attention and eye movements. Neuron 21, 761–773. 10.1016/S0896-6273(00)80593-0 9808463

[B12] Craighero L. (2014). “The role of the motor system in cognitive functions,” in The Routledge Handbook of Embodied Cognition, ed. Shapiro L. (New York: Routledge), 51–58.

[B13] Craighero L. Bonetti F. Massarenti L. Canto R. Fabbri Destro M. Fadiga L. (2008). Temporal prediction of touch instant during observation of human and robot grasping. Brain Res. Bull. 75, 770–774. 10.1016/j.brainresbull.2008.01.014 18394523

[B14] Craighero L. Fadiga L. Rizzolatti G. Umiltà C. A. (1998). Visuomotor priming. Vis. Cogn. 5, 109–125. 10.1080/713756780 9051808

[B15] Craighero L. Fadiga L. Rizzolatti G. Umiltà C. A. (1999). Action for perception: a motor-visual attentional effect. J. Exp. Psychol. Hum. Percept. Perform. 25, 1673–1692. 10.1037/0096-1523.25.6.1673 10641315

[B16] Craighero L. Fadiga L. Umiltà C. A. Rizzolatti G. (1996). Evidence for visuomotor priming effect. Neuroimage 8, 347–349. 10.1097/00001756-199612200-00068 9051808

[B17] Craighero L. Zorzi V. (2012). Hand-foot motor priming in the presence of temporary inability to use hands. Vis. Cogn. 20, 77–93. 10.1080/13506285.2011.639315 22577335 PMC3337042

[B18] Craighero L. Zorzi V. Canto R. Franca M. (2014). Same kinematics but different objects during action observation: detection times and motor evoked potentials. Vis. Cogn. 22, 653–671. 10.1080/13506285.2014.904460

[B19] Creem-Regehr S. H. Lee J. N. (2005). Neural representations of graspable objects: are tools special? Brain Res. Cogn. Brain Res. 22, 457–469. 10.1016/j.cogbrainres.2004.10.006 15722215

[B20] D’Ausilio A. Jarmolowska J. Busan P. Bufalari I. Craighero L. (2011). Tongue corticospinal modulation during attended verbal stimuli: priming and coarticulation effects. Neuropsychologia 49, 3670–3676. 10.1016/j.neuropsychologia.2011.09.022 21958646

[B21] Desmurget M. Grafton S. (2000). Forward modeling allows feedback control for fast reaching movements. Trends Cogn. Sci. 4, 423–431. 10.1016/S1364-6613(00)01537-0 11058820

[B22] Di Pellegrino G. Fadiga L. Fogassi L. Gallese V. Rizzolatti G. (1992). Understanding motor events: a neurophysiological study. Exp. Brain Res. 91, 176–180. 10.1007/BF00230027 1301372

[B23] Ekstrom L. B. Roelfsema P. R. Arsenault J. T. Bonmassar G. Vanduffel W. (2008). Bottom-up dependent gating of frontal signals in early visual cortex. Science 321, 414–417. 10.1126/science.1153276 18635806 PMC3011100

[B24] Ellis R. Tucker M. (2000). Micro-affordance: the potentiation of components of action by seen objects. Br. J. Psychol. 9, 451–471. 10.1348/000712600161934 11104173

[B25] Fadiga L. Fogassi L. Pavesi G. Rizzolatti G. (1995). Motor facilitation during action observation: a magnetic stimulation study. J. Neurophysiol. 73, 2608–2611. 7666169 10.1152/jn.1995.73.6.2608

[B26] Fagioli S. Hommel B. Schubotz R. I. (2007). Intentional control of attention: action planning primes action-related stimulus dimensions. Psychol. Res. 71, 22–29. 10.1007/s00426-005-0033-3 16317565

[B27] Fazio P. Cantagallo A. Craighero L. D’Ausilio A. Roy A. C. Pozzo T. (2009). Encoding of human action in Broca’s area. Brain 132, 1980–1988. 10.1093/brain/awp118 19443630

[B28] Fieandt K. Huhtala A. Kullberg P. Saarl K. (1956). Personal Tempo and Phenomenal Time at Different Age Levels. Reports From the Psychological Institute, No. 2, Helsinki: University of Helsinki.

[B29] Fischer M. H. Hoellen N. (2004). Space-based and object-based attention depend on motor intention. J. Gen. Psychol. 131, 365–377. 15523820

[B30] Flanagan J. R. Johansson R. S. (2003). Action plans used in action observation. Nature 424, 769–771. 10.1038/nature01861 12917683

[B31] Flanagan J. R. Rotman G. Reichelt A. F. Johansson R. S. (2013). The role of observers’ gaze behaviour when watching object manipulation tasks: predicting and evaluating the consequences of action. Philos. Trans. R. Soc. Lond. B Biol. Sci. 368, 20130063. 10.1098/rstb.2013.0063 24018725 PMC3758206

[B32] Gallese V. Fadiga L. Fogassi L. Rizzolatti G. (1996). Action recognition in the premotor cortex. Brain 119, 593–609. 10.1093/brain/119.2.593 8800951

[B33] Galton F. (1899). On instruments for (1) testing perception of differences of tint and for (2) determining reaction time. J. Anthropol. Inst. 19, 27–29.

[B34] Gangitano M. Mottaghy F. M. Pascual-Leone A. (2001). Phase-specific modulation of cortical motor output during movement observation. Neuroimage 12, 1489–1492. 10.1097/00001756-200105250-00038 11388435

[B35] Gangitano M. Mottaghy F. M. Pascual-Leone A. (2004). Modulation of premotor mirror neuron activity during observation of unpredictable grasping movements. Eur. J. Neurosci. 20, 2193–2202. 10.1111/j.1460-9568.2004.03655.x 15450099

[B36] Gerlach C. Law I. Paulson O. B. (2002). When action turns into words. Activation of motor-based knowledge during categorization of manipulable objects. J. Cogn. Neurosci. 14, 1230–1239. 10.1162/089892902760807221 12495528

[B37] Grafton S. T. Fadiga L. Arbib M. A. Rizzolatti G. (1997). Premotor cortex activation during observation and naming of familiar tools. Neuroimage 6, 231–236. 10.1006/nimg.1997.0293 9417966

[B38] Grezes J. Decety J. (2002). Does visual perception of object afford action? Evidence from a neuroimaging study. Neuropsychologia 4, 212–222. 10.1016/S0028-3932(01)00089-6 11640943

[B39] Grezes J. Tucker M. Armony J. Ellis R. Passingham R. E. (2003). Objects automatically potentiate action: an fMRI study of implicit processing. Eur. J. Neurosci. 17, 2735–2740. 10.1046/j.1460-9568.2003.02695.x 12823480

[B40] Hannus A. Cornelissen F. W. Lindemann O. Bekkering H. (2005). Selection-for-action in visual search. Acta Psychol. 118, 171–191. 10.1016/j.actpsy.2004.10.010 15627415

[B41] Janet P. (1935). Les Débuts de l’intelligence. Paris: Flammarion.

[B42] Jeannerod M. (1995). Mental imagery in the motor context. Neuropsychologia 33, 1419–1432. 10.1016/0028-3932(95)00073-C 8584178

[B43] Klatzky R. L. Fikes T. G. Pellegrino J. W. (1995). Planning for hand shape and arm transport when reaching for objects. Acta Psychol. (Amst.) 88, 209–232. 10.1016/0001-6918(93)E0068-D 7597925

[B44] Lotze R. H. (1852). Medizinische Psychologie oder Physiologie der Seele. Leipzig: Weidemann.

[B45] Martin A. Wiggs C. L. Ungerleider L. G. Haxby J. V. (1996). Neural correlates of category-specific knowledge. Nature 379, 649–652. 10.1038/379649a0 8628399

[B46] Matelli M. Luppino G. (2001). Parietofrontal circuits for action and space perception in the macaque monkey. Neuroimage 14(1 Pt 2), S27–S32. 10.1006/nimg.2001.0835 11373129

[B47] Montagna M. Cerri G. Borroni P. Baldissera F. (2005). Excitability changes in human corticospinal projections to muscles moving hand and fingers while viewing a reaching and grasping action. Eur. J. Neurosci. 22, 1513–1520. 10.1111/j.1460-9568.2005.04336.x 16190904

[B48] Moore T. Armstrong K. M. (2003). Selective gating of visual signals by microstimulation of frontal cortex. Nature 421, 370–373. 10.1038/nature01341 12540901

[B49] Moore T. Fallah M. (2001). Control of eye movements and spatial attention. Proc. Natl. Acad. Sci. U.S.A. 98, 1273–1276. 10.1073/pnas.98.3.1273 11158629 PMC14744

[B50] Murata A. Fadiga L. Fogassi L. Gallese V. Raos V. Rizzolatti G. (1997). Object representation in the ventral premotor cortex (area F5) of the monkey. J. Neurophysiol. 78, 2226–2230. 9325390 10.1152/jn.1997.78.4.2226

[B51] Nobre A. C. Gitelman D. R. Dias E. C. Mesulam M. M. (2000). Covert visual spatial orienting and saccades: overlapping neural systems. Neuroimage 11, 210–216. 10.1006/nimg.2000.0539 10694463

[B52] Noppeney U. Josephs O. Kiebel S. Friston K. J. Price C. J. (2005). Action selectivity in parietal and temporal cortex. Brain Res. Cogn. Brain Res. 25, 641–649. 10.1016/j.cogbrainres.2005.08.017 16242924

[B53] Okada T. Tanaka S. Nakai T. Nishizawa S. Inui T. Sadato N. (2000). Naming of animals and tools: a functional magnetic resonance imaging study of categorical differences in the human brain areas commonly used for naming visually presented objects. Neurosci. Lett. 296, 33–36. 10.1016/S0304-3940(00)01612-8 11099827

[B54] Oldfield R. C. (1971). The assessment and analysis of handedness: the Edinburgh inventory. Neuropsychologia 9, 97–113. 10.1016/0028-3932(71)90067-4 5146491

[B55] Posner M. I. Nissen M. J. Ogden W. C. (1978). “Attended and unattended processing modes: the role of set for spatial location,” in Modes of Perceiving and Processing Information, eds Pick H. L. Saltzman E. J. (Hillsdale, NJ: Erlbaum), 137–157.

[B56] Raos V. Umiltá M. A. Murata A. Fogassi L. Gallese V. (2006). Functional properties of grasping-related neurons in the ventral premotor area F5 of the macaque monkey. J. Neurophysiol. 95, 709–729. 10.1152/jn.00463.2005 16251265

[B57] Rizzolatti G. Craighero L. (1998). “Spatial attention: mechanisms and theories,” in Advances in Psychological Science, Vol. 2, *Biological and Cognitive Aspects*, eds Sabourin M. Craik F. Robert M. (East Sussex: Psychology Press), 171–198.

[B58] Rizzolatti G. Craighero L. (2004). The mirror-neuron system. Annu. Rev. Neurosci. 27, 169–192. 10.1146/annurev.neuro.27.070203.144230 15217330

[B59] Rizzolatti G. Fadiga L. Fogassi L. Gallese V. (1997). The space around us. Science 277, 190–191. 10.1126/science.277.5323.190 9235632

[B60] Rizzolatti G. Fadiga L. Gallese V. Fogassi L. (1996). Premotor cortex and the recognition of motor actions. Cogn. Brain Res. 3, 131–141. 10.1016/0926-6410(95)00038-0 8713554

[B61] Rizzolatti G. Riggio L. Dascola I. Umiltà C. (1987). Reorienting attention across the horizontal and vertical meridians: evidence in favor of a premotor theory of attention. Neuropsychologia 25, 31–40. 10.1016/0028-3932(87)90041-8 3574648

[B62] Rizzolatti G. Riggio L. Sheliga B. M. (1994). “Space and selective attention” in Attention and Performance XV, eds Umiltà C. Moscovitch M. (Cambridge, MA: MIT Press), 231–265.

[B63] Ruff C. C. Blankenburg F. Bjoertomt O. Bestmann S. Freeman E. Haynes J. D. (2006). Concurrent TMS-fMRI and psychophysics reveal frontal influences on human retinotopic visual cortex. Curr. Biol. 16, 1479–1488. 10.1016/j.cub.2006.06.057 16890523

[B64] Symes E. Tucker M. Ellis R. Vainio L. Ottoboni G. (2008). Grasp preparation improves change detection for congruent objects. J. Exp. Psychol. Hum. Percept. Perform. 34, 854–871. 10.1037/0096-1523.34.4.854 18665731

[B65] Theeuwes J. (2013). Feature based attention: it is all bottom-up priming. Philos. Trans R. Soc. Lond. B Biol. Sci. 368, 20130055. 10.1098/rstb.2013.0055 24018717 PMC3758198

[B66] Tucker M. Ellis R. (1998). On the relations of seen objects and components of potential actions. J. Exp. Psychol. Hum. Percept. Perform. 24, 830–846. 10.1037/0096-1523.24.3.830 9627419

[B67] Tucker M. Ellis R. (2001). The potentiation of grasp types during visual object categorization. Vis. Cogn. 8, 769–800. 10.1080/13506280042000144

[B68] Tucker M. Ellis R. (2004). Action priming by briefly presented objects. Acta Psychol. (Amst.) 116, 185–203. 10.1016/j.actpsy.2004.01.004 15158182

[B69] von Wright J. M. (1970). On selection in visual immediate memory. Acta Psychol. 33, 280–292. 10.1016/0001-6918(70)90140-X 5445966

[B70] Welford A. T. (1980). “Choice reaction time: basic concepts,” in Reaction Times, ed. Welford A. T. (New York: Academic Press), 73–128.

